# Crystal structure and Hirshfeld surface analysis of (2,7-di­eth­oxy­naphthalene-1,8-di­yl)bis­[(4-bromophen­yl)methanone]

**DOI:** 10.1107/S205698902401123X

**Published:** 2024-11-22

**Authors:** Kun Li, Jiali Yao, Hiroaki Iitsuka, Noriyuki Yonezawa, Akiko Okamoto

**Affiliations:** a Department of Organic and Polymer Materials Chemistry Tokyo University of Agriculture and Technology 2-24-16 Nakamachi Koganei Tokyo 184-8588 Japan; Tokyo University of Science, Japan

**Keywords:** non-coplanar aromatic rings, symmetric spatial organization, non-classical hydrogen bonds, bromo⋯hydrogen/carbon short contacts, Hirshfeld surface analysis, crystal structure

## Abstract

The title compound, C_28_H_22_Br_2_O_4_, crystallizes in the monoclinic space group *P*2_1_/*c* at 193 K. The two 4-bromo­benzoyl groups are attached in a non-coplanar fashion to the naphthalene ring system and are oriented in opposite directions. The two 4-bromo­benzene rings are tilted almost symmetrically with respect to the naphthalene ring system. The crystal packing features several kinds of non-classical hydrogen bonds such as C—H⋯*X* (*X* = polar atom), in which the C—H group acts as a hydrogen-atom donor, and C—H⋯π inter­actions. The two-dimensional fingerprint plots generated in a Hirshfeld surface analysis show non-classical hydrogen bonding with short contacts involving the bromo group.

## Abstract 2

In the title compound, the two 4-bromo­benzoyl groups are attached in a non-coplanar fashion to the naphthalene ring system and are oriented in opposite directions.

## 1. Chemical context

Supra­molecular architectures along with supra­molecular chemistry have become of inter­est in recent years from the viewpoint of green chemistry and novel phases of functional device material development (Desiraju, 1989 ▸; Lehn, 1995 ▸; Atwood *et al.*, 1996 ▸; Desiraju *et al.*, 2011 ▸). Various building blocks bearing unique functions might be tailored to a supra­molecular structure exhibiting desired chemical and physical properties without formation of covalent bonds. The research primarily relies on knowledge of the characteristics of non-covalent bonding inter­actions, including atomic, geometrical and mol­ecular orientation features (Jeffrey & Saenger, 1991 ▸; Steiner & Desiraju, 1998 ▸). Attempts to form robust hydrogen bonds involving CON*R*_2_ and OH groups, and COOH and NH_2_ groups were undertaken both experimentally and theoretically. These have been successfully employed for the preparation of numerous mol­ecular assemblies (Price, 2004 ▸; Tanabe *et al.*, 2013 ▸; Hubbard *et al.*, 2016 ▸). On the other hand, attempts to grasp the nature of weak hydrogen bonds, including non-classical hydrogen bonds where the C—H group acts as a hydrogen-atom donor, for example, have scarcely been achieved, probably because they are often hidden by strong hydrogen bonds. Congested mol­ecules of accumulated aromatic rings have unique spatial structural restrictions such that the aromatic rings are compelled to be arranged in a non-coplanar manner. This structural constraint suggests that the contribution of a parallel overlap of aromatic rings, *i.e.*, π–π stacking is extremely small. From this viewpoint, the crystal structures of compounds with non-coplanarly accumulated aromatic rings can be expected to emphasize the contributions of rather weak, non-covalent bonding inter­actions other than π–π stacking inter­actions and classical hydrogen bonds. The authors have studied compounds with non-coplanarly accumulated aromatic ring structures in formation reactions and spatial structures (Okamoto & Yonezawa, 2009 ▸, 2015 ▸; Okamoto *et al.*, 2011 ▸).

*peri*-Aroyl­naphthalene compounds and their homologues usually show an excellent tendency to give single crystallinity, and the crystal structures of over 100 homologues and related compounds have been determined by the authors. With the aid of a systematical comparison of the single mol­ecular structure and accumulation fashion of the crystal structure of series of homologous compounds, the transition of the inter­action feature among the homologues has been clarified, which suggests important roles for weak inter­actions involving C—H hydrogen atoms in the determination of the crystalline spatial placement of mol­ecules (Iida *et al.*, 2022 ▸; Kobayashi *et al.*, 2023 ▸). Herein, the authors report on the crystal structure and Hirshfeld surface analysis of the title *peri*-aroyl­naphthalene, (2,7-di­eth­oxy­naphthalene-1,8-di­yl)bis­[(4-bromo­phen­yl)methanone].
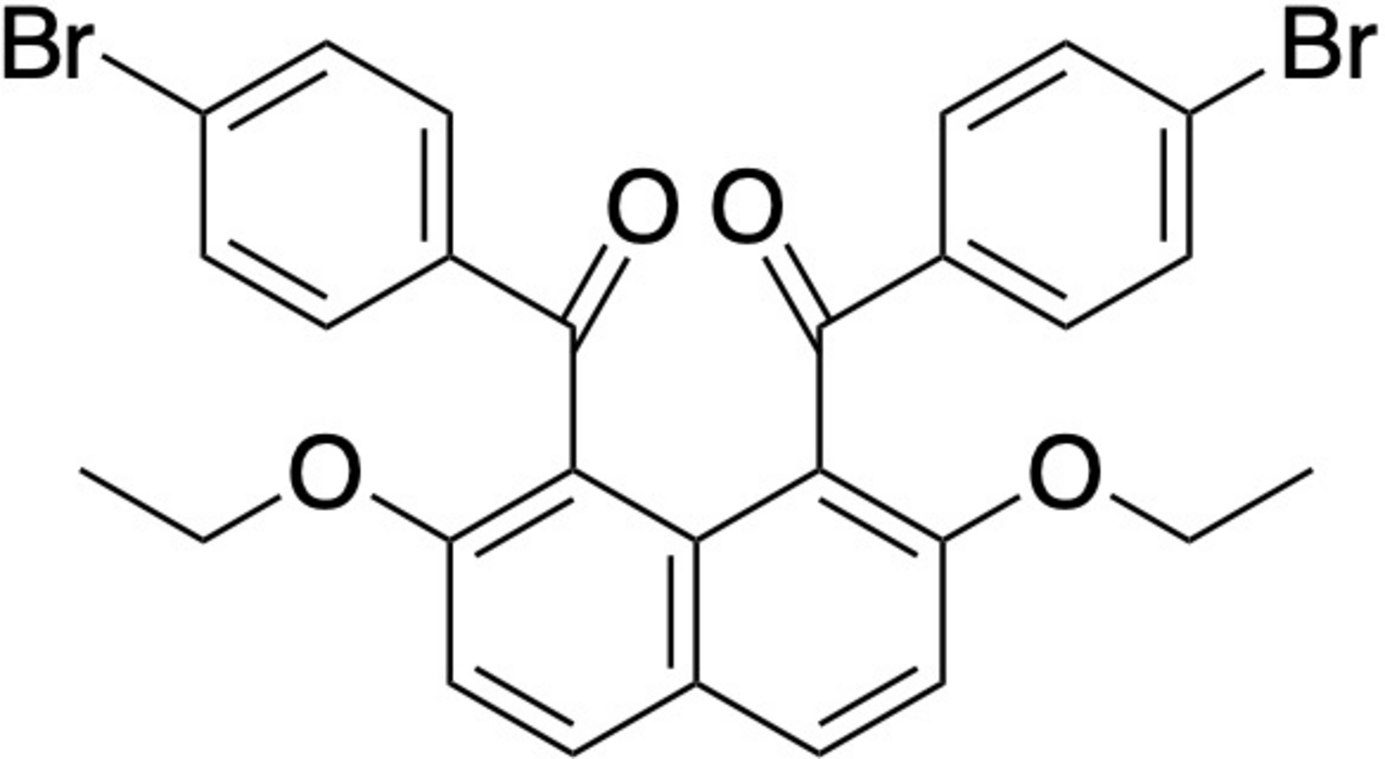


## 2. Structural commentary

**Figure 1:**
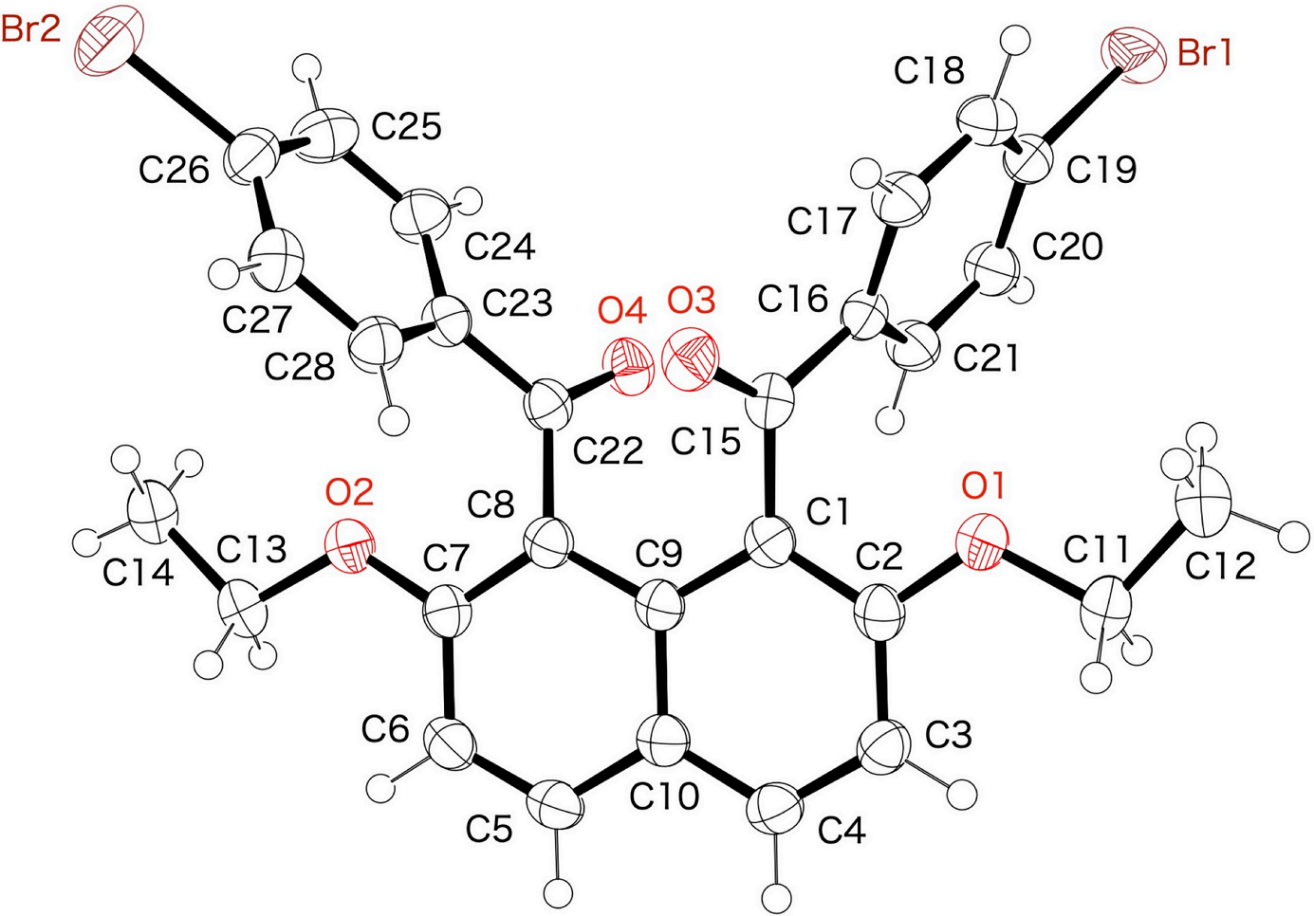
Mol­ecular structure of the title compound with displacement ellipsoids at the 50% probability level.

The mol­ecular structure of the title mol­ecule is displayed in Fig. 1 ▸. The two 4-bromo­benzoyl groups are twisted with respect to the naphthalene ring system and oriented in opposite direction (*anti* orientation). The two 4-bromo­benzene rings are tilted almost symmetrically to the naphthalene ring system. The two inter­planar angles between the best planes of the 4-bromo­benzene rings and the naphthalene ring system are 79.38 (10) and 79.50 (10)°, respectively [torsion angles: C9—C1—C15—O3 = 54.9 (3) and C9—C8—C22—O4 = 50.5 (3)°]. On the other hand, the 4-bromo­benzene rings are coplanar with the carbonyl moieties [torsion angles: O3—C15—C16—C21 = −155.7 (2) and O4—C22—C23—C28 = −155.5 (2)°]. The inter­planar angle between the best planes of the two 4-bromo­benzene rings is 60.44 (12)°.

## 3. Supra­molecular features

**Figure 2:**
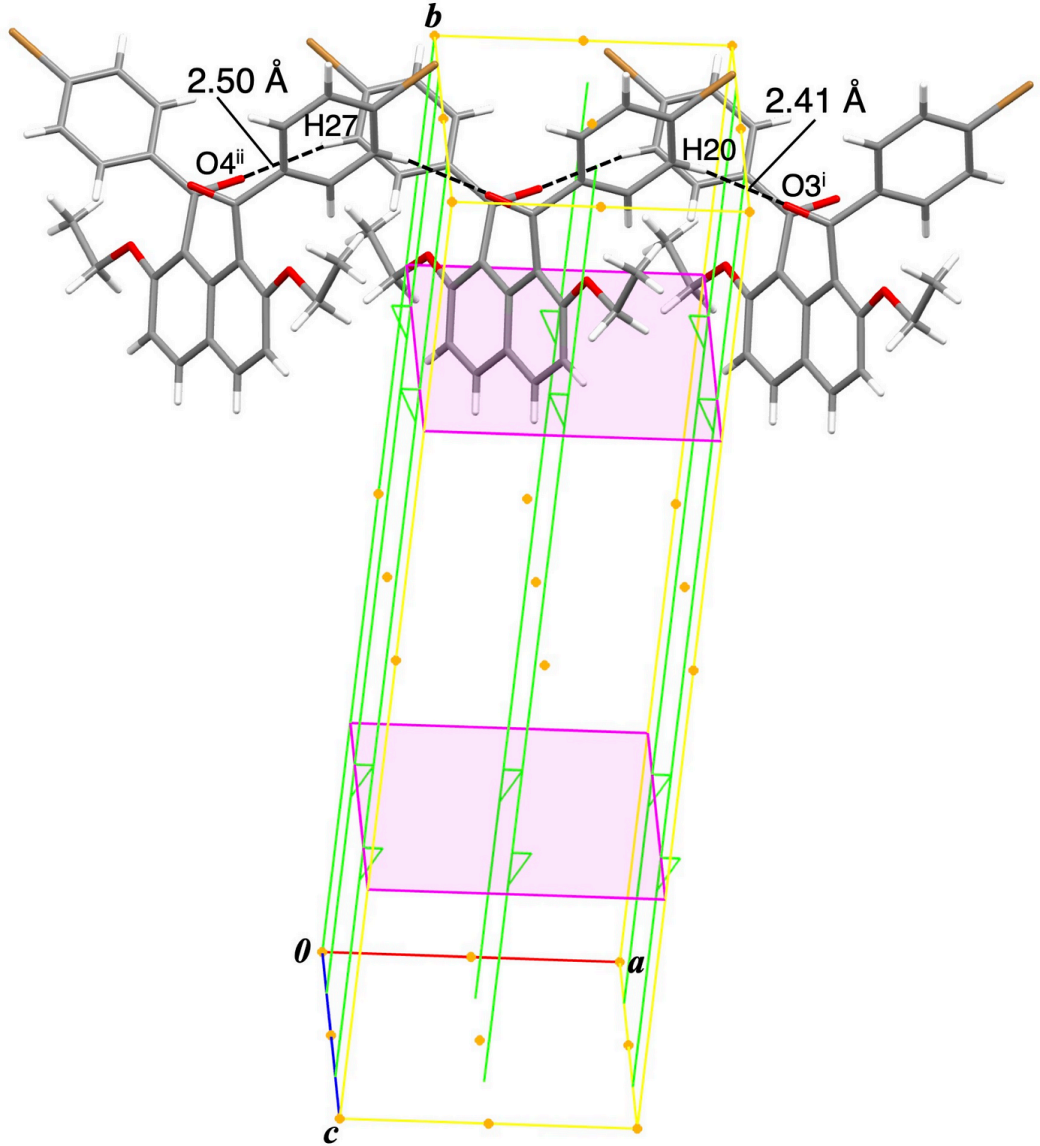
Mol­ecular packing structure of the title compound showing the non-covalent bonding inter­actions and the relationship with the symmetry elements: C—H⋯O non-classical hydrogen bonds connect the mol­ecules in a translational relationship along *a*-axis [symmetry codes: (i)1 + *x*, *y*, *z*; (ii) −1 + *x*, *y*, *z*].

**Figure 3:**
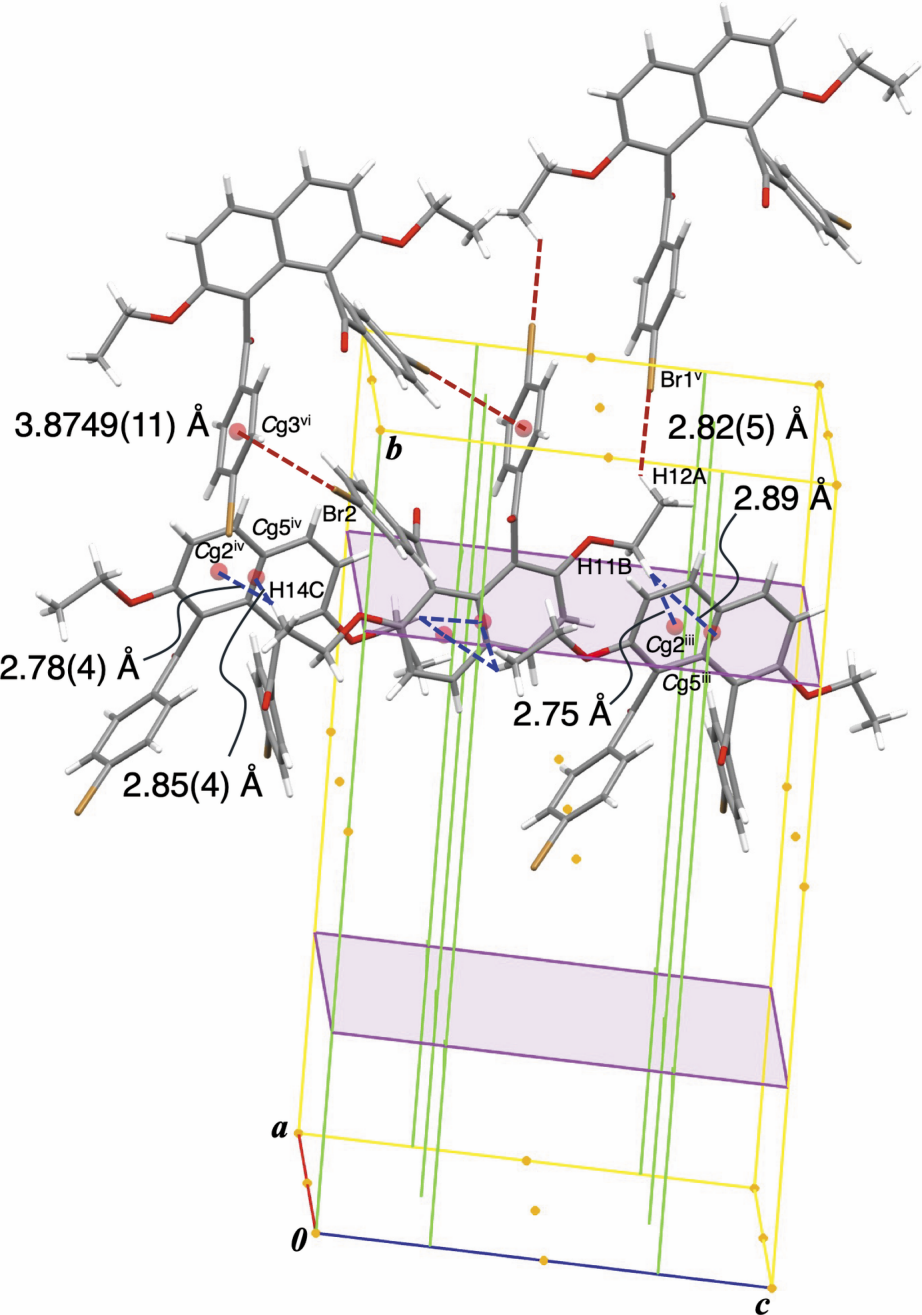
Crystal packing structure of title compound showing non-covalent bonding inter­actions and the relationship with symmetry elements. C—H⋯π non-classical hydrogen bonds connect the mol­ecules along the *ac*-glide plane [symmetry codes: (iii) *x*, 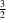 − *y*, 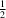 + *z*; (iv) *x*, 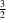 − *y*, −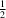 + *z*]. C—H⋯Br non-classical hydrogen bonds and C—Br⋯π short contacts link pairs of mol­ecules centrosymmetrically along the *b*-axis [symmetry codes: (v) 2 − *x*, 2 − *y*, 1 − *z*; (vi) −*x*, 2 − *y*, −*z*].

In the crystal, several kinds of non-classical hydrogen bonds ensure the cohesion of the packing. C—H⋯O=C non-classical hydrogen bonds between the 4-bromo­benzene ring and the carbonyl O atom arrange the mol­ecules in a translational relationship along *a*-axis direction [C20—H20⋯O3^i^ = 2.41 Å, C27—H27⋯O4^ii^ = 2.50 Å; symmetry codes: (i) 1 + *x*, *y*, *z*; (ii) −1 + *x*, *y*, *z*] (Table 1 ▸, Fig. 2 ▸). C—H⋯π non-classical hydrogen bonds between the eth­oxy moiety and the naphthalene ring system connect the mol­ecules along *ac*-glide plane [C11—H11*B*⋯*Cg*2^iii^ = 2.75 Å, C11—H11*B*⋯*Cg*5^iii^ = 2.89 Å, C14—H14*C*⋯*Cg*2^iv^ = 2.78 (4) Å, C14—H14*C*⋯*Cg*5^iv^ = 2.85 (4) Å; symmetry codes: (iii) *x*, 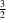 − *y*, 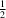 + *z*; (iv) *x*, 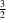 − *y*, −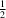 + *z*; *Cg*2 and *Cg*5 are the centroids of the C5–C10 ring and the C1–C10 ring system, respectively] (Fig. 3 ▸). The naphthalene ring system acts as hydrogen-atom acceptor of dual C*sp*^3^—H⋯π non-classical hydrogen bonds. Inter­actions involving the bromo group are formed complimentarily between two mol­ecules, forming centrosymmetric dimeric aggregations [C12—H12*A*⋯Br1^v^ = 2.82 (5) Å, C26—Br2⋯*Cg*3^vi^ = 3.8749 (11) Å; symmetry codes: (v) 2 − *x*, 2 − *y*, 1 − *z*; (vi) −*x*, 2 − *y*, −*z*; *Cg*3 is the centroid of the C16–C21 ring] (Fig. 3 ▸).

## 4. Database survey

A search of the Cambridge Structural Database (CSD, Version 5.45, update of November 2023; Groom *et al.*, 2016 ▸) for the 1,8-di­benzoyl­naphthalene and 1,8-diaroyl-2,7-di­alk­oxy­naphthalene frameworks of the title compound yields 39 and 29 hits, respectively. The structure of the title compound exhibits non-coplanarly accumulated aromatic rings, as found in the bromo group-free 1,8-di­benzoyl­naphthalene homologues, the bromo group-bearing 1,8-di­benzoyl­naphthalene, and 1-benzoyl­naphthalene homologues, *viz.* 1,8-dibenzoyl-2,7-di­meth­oxy­naphthalene (CSD refcode XIYSEE: Nakaema *et al.*, 2008 ▸), 1,8-dibenzoyl-2,7-di­eth­oxy­naphthalene (CSD refcode NEQRUY; Isogai *et al.*, 2013 ▸), 1,8-bis­(4-bromo­benzo­yl)-2,7-di­meth­oxy­naphthalene (CSD refcode DUNRUA; Watanabe *et al.*, 2010 ▸), (8-[4-(bromo­meth­yl)benzo­yl]-2,7-di­meth­oxy­naphthalen-1-yl)[4-(bromo­meth­yl)phen­yl]methanone (CSD refcode EVIWUC; Sasagawa *et al.*, 2011 ▸), (2,7-di­meth­oxy­naphthalen-1-yl)(phen­yl)methanone (CSD refcode KABGAX; Kato, Nagasawa, Hijikata *et al.*, 2010 ▸), and 2,7-dimeth­oxy-1-(4-bromo­benzo­yl)naphthalene (CSD refcode VACLIW; Kato, Nagasawa, Tsumuki *et al.*, 2010 ▸). The dihedral angle between the benzene ring and the naphthalene ring system in 1,8-dibenzoyl-2,7-di­meth­oxy­naphthalene (XIYSEE) is larger than in the 2,7-dieth­oxy homologues (NEQRUY), *i.e.*, 83.59 (5)° *vs* 68.42 (5) and 71.69 (5)°. On the other hand, the 4-bromo­benzoyl group-bearing homologues exhibit the opposite tendency, *viz.* 70.18 (11) and 74.98 (12)° for 1,8-bis­(4-bromo­benzo­yl)-2,7-di­meth­oxy­naphthalene (DUNRUA) *vs* 79.38 (10) and 79.50 (10)° for the title compound. The homologue bearing a bromo group bonded to an *sp*^3^ carbon atom (EVIWUC) has almost the same dihedral angles as the 2,7-di*meth­oxy*naphthalene homologue (DUNRUA) [70.98 (13) and 72.89 (13)°]. The 1-benzoyl­ated homologue (KABGAX) affords three conformers, which have different dihedral angles between the benzene ring and the naphthalene ring system [75.34 (7), 86.47 (7) and 76.55 (6)°]. The homologue with a bromo group gives solely one type of conformer, even if it is 1-monoaroylated homologue [VACLIW; 72.02 (9)°]. Therefore, the introduction of a bromo group at the benzoyl groups at the 1,8-positions has a larger effect on the dihedral angle than an eth­oxy group at the 2,7-positions. Furthermore, intra/inter­molecular inter­actions involving the bromo groups contribute significantly to the three-dimensional mol­ecular structure and packing structure.

## 5. Hirshfeld surface analysis and two-dimensional fingerprint plots

**Figure 4:**
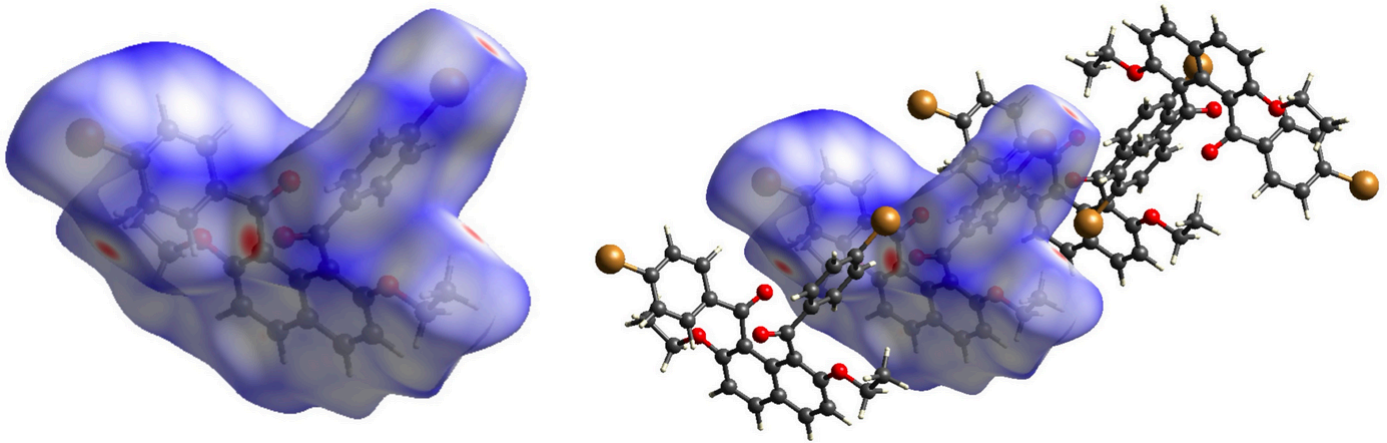
The Hirshfeld surface of the title compound mapped over *d*_norm_ (left). Several large red points are assigned as O3⋯H20, O5⋯H27, and Br1⋯H12*A* short contacts (right).

The Hirshfeld surface analysis (Spackman & Jayatilaka, 2009 ▸) was performed and the associated two-dimensional fingerprint plots (McKinnon *et al.*, 2007 ▸) were generated with *CrystalExplorer17* (Turner *et al.*, 2017 ▸). The Hirshfeld surfaces are colour-mapped with the normalized contact distance, *d*_norm_, from red (distances shorter than the sum of the van der Waals radii) through white to blue (distances longer than the sum of the van der Waals radii). The Hirshfeld surface of the title compound mapped over *d*_norm_ in the range −0.2470 to 1.3548 a.u. is shown in Fig. 4 ▸ (left). The red points represent close contacts and negative *d*_norm_ values on the surface. Several large red points correspond to the short contacts involving the carbonyl O atoms, O3^i^ and O4^ii^, and hydrogen atoms, H20 and H27 [symmetry codes: (i)1 + *x*, *y*, *z*; (ii) −1 + *x*, *y*, *z*], and short Br1^v^⋯H12*A* inter­actions [symmetry code: (v) 2 − *x*, 2 − *y*, 1 − *z*] (Fig. 4 ▸, right).

**Figure 5:**
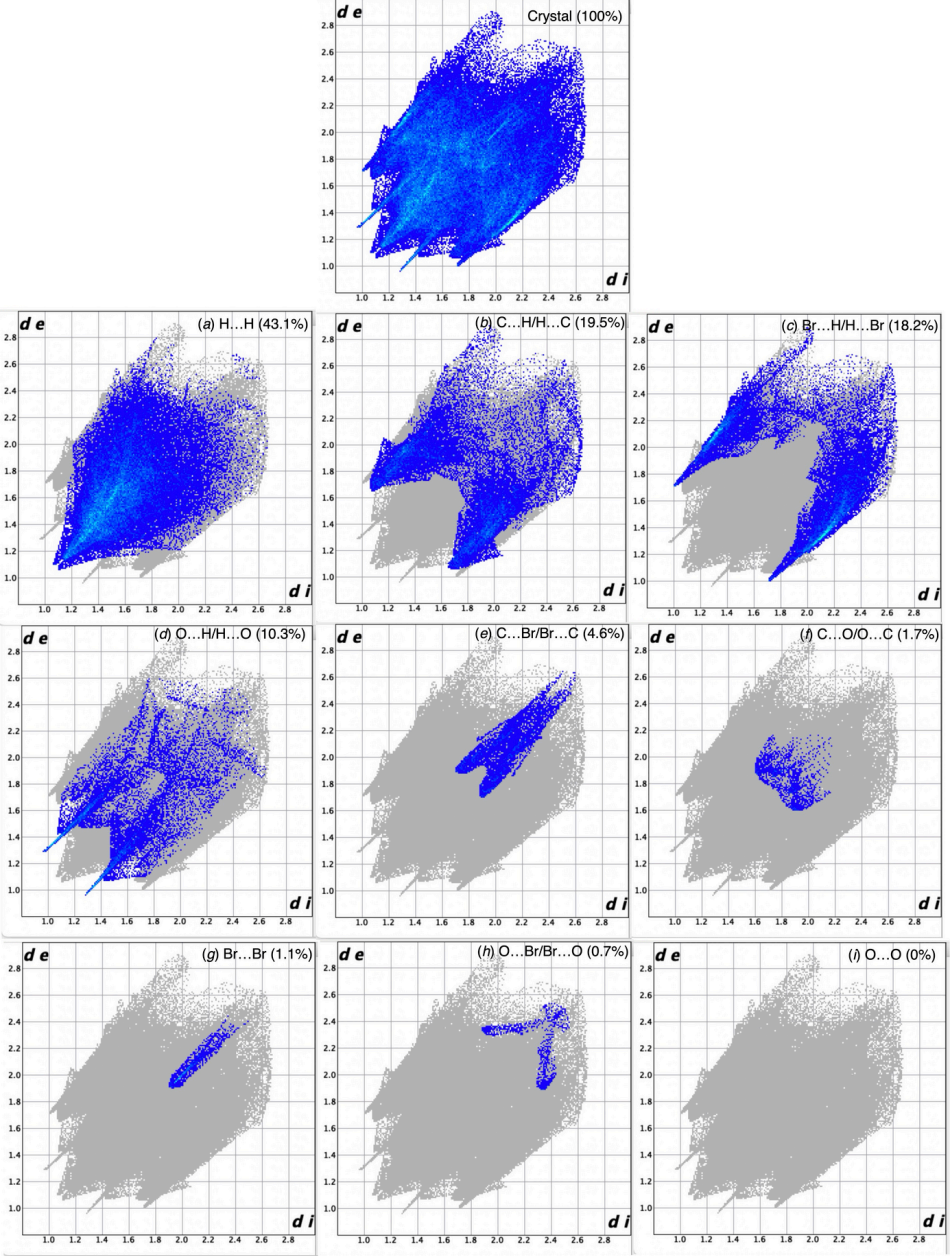
The full two-dimensional fingerprint plot for the title compound, and those delineated into (*a*) H⋯H, (*b*) C⋯H/H⋯C, (*c*) Br⋯H/H⋯Br, (*d*) O⋯H/H⋯O, (*e*) C⋯Br/Br⋯C, (*f*) C⋯O/O⋯C, (*g*) Br⋯Br, (*h*) O⋯Br/Br⋯O, and (*i*) O⋯O contacts.

The two-dimensional fingerprint plots from the Hirshfeld surface analysis are shown in Fig. 5 ▸, revealing the inter­molecular contacts and their percentage contributions to the Hirshfeld surface. Not surprisingly, H⋯H contacts are the major contributor (43.1%), while the Br⋯H/H⋯Br contacts (18.2%) are present as a relatively large contributor, on the same level as C⋯H/H⋯C contacts (19.5%). O⋯H/H⋯O (10.3%), C⋯Br/Br⋯C (4.6%), C⋯O/O⋯C (1.7%), Br⋯Br (1.1%), and O⋯Br/Br⋯O (0.7%) contacts also make significant contributions to the Hirshfeld surface.

## 6. Synthesis and crystallization

To a 10 ml flask, 4-bromo­benzoyl chloride (6.0 mmol, 1.32 g), TiCl_4_ (18 mmol, 3.41 g) and methyl­ene dichloride (3.6 ml) were placed and stirred at 273 K. To reaction mixture thus obtained, 2,7-di­eth­oxy­naphthalene (2.0 mmol, 433 mg) was added. After the reaction mixture was stirred at 298 K for 24 h, it was poured into ice-cold water (30 ml). The aqueous layer was extracted with CHCl_3_ (20 ml, three times). The combined extracts were washed with 2 *M* aqueous NaOH followed by washing with brine. The organic layers thus obtained were dried over anhydrous MgSO_4_. The solvent was removed under reduced pressure to give a cake (96% crude yield). The crude product was purified by reprecipitation (CHCl_3_/methanol; isolated yield 77%). Finally, the isolated product was crystallized from methanol to give single crystals.

^1^H NMR (300 MHz, CDCl_3_): 0.962 (6H, *t*, *J* = 6.9 Hz), 3.97 (4H, *q*, *J* = 6.9 Hz), 7.16 (2H, *d*, *J* = 9.0 Hz), 7.50 (4H, *d*, *J* = 8.1 Hz), 7.59 (4H, *d*, *J* = 8.7 Hz), 7.93 (2H, *d*, *J* = 9.0 Hz) ppm; ^13^C NMR (75 MHz, CDCl_3_): 14.5, 65.0, 112.19, 121.02, 125.53, 127.67, 130.42, 130.58, 131.35, 132.54, 138.17, 156.13, 196.83 ppm; IR (KBr): 1658 (C=O), 1608, 1584, 1509 (Ar, naphthalene), 1274, 1112 (C—O—C) cm^−1^; HRMS (FAB): calculated for C_28_H_23_Br_2_O_4_ [*M* + H]^+^, 580.9958, found, 580.9963; m.p. = 491–492 K.

## 7. Refinement

Crystal data, data collection and structure refinement details are summarized in Table 2 ▸. All H atoms were located in difference-Fourier maps and were subsequently refined as riding atoms, with C—H = 0.95 (aromatic), 0.98 (meth­yl) and 0.99 Å (methyl­ene), and with *U*_iso_(H) = 1.2*U*_eq_(C). The positions of the methyl H atoms were rotationally optimized.

## Supplementary Material

Crystal structure: contains datablock(s) I. DOI: 10.1107/S205698902401123X/jp2015sup1.cif

Structure factors: contains datablock(s) I. DOI: 10.1107/S205698902401123X/jp2015Isup2.hkl

Spectral data. DOI: 10.1107/S205698902401123X/jp2015sup3.pdf

CCDC reference: 2403767

Additional supporting information:  crystallographic information; 3D view; checkCIF report

## Figures and Tables

**Table 1:** Hydrogen-bond geometry (Å, °) *Cg*2, *Cg*3 and *Cg*5 are the centroids of the C5–C10, C16–C21 and C1–C10 rings, respectively.

*D*—H⋯*A*	*D*—H	H⋯*A*	*D*⋯*A*	*D*—H⋯*A*
C20—H20⋯O3^i^	0.95	2.41	3.339 (3)	167
C27—H27⋯O4^ii^	0.95	2.50	3.439 (3)	170
C11—H11*B*⋯*Cg*2^iii^	0.99	2.75	3.632 (3)	148
C11—H13*B*⋯*Cg*5^iii^	0.99	2.89	3.838 (3)	162
C14—H14*C*⋯*Cg*2^iv^	0.98	2.78 (4)	3.631 (3)	151 (3)
C14—H14*C*⋯*Cg*5^iv^	0.98	2.85 (4)	3.632 (3)	141 (3)
C12—H12*A*⋯Br1^v^	0.97 (4)	2.82 (5)	3.634 (4)	141 (3)
C26—Br2⋯*Cg*3^vi^	1.90 (1)	3.88 (1)	5.626 (3)	152 (1)

Symmetry codes: (i) 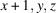; (ii) 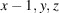; (iii) 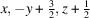; (iv) 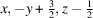; (v) 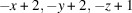; (vi) 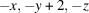.

**Table 2:** Experimental details

Crystal data	
Chemical formula	C_28_H_22_Br_2_O_4_
*M*_r_	582.27
Crystal system, space group	Monoclinic, *P*2_1_/*c*
Temperature (K)	193
*a*, *b*, *c* (Å)	7.74560 (14), 24.4234 (4), 13.0590 (2)
β (°)	101.6109 (9)
*V* (Å^3^)	2419.86 (8)
*Z*	4
Radiation type	Cu *K*α
μ (mm^−1^)	4.52
Crystal size (mm)	0.60 × 0.30 × 0.10

Data collection	
Diffractometer	Rigaku R-AXIS RAPID
Absorption correction	Numerical (*NUMABS*; Rigaku, 1999 ▸)
*T*_min_, *T*_max_	0.260, 0.636
No. of measured, independent and observed [*I* > 2σ(*I*)] reflections	43148, 4414, 4038
*R*_int_	0.080
(sin θ/λ)_max_ (Å^−1^)	0.602

Refinement	
*R*[*F*^2^ > 2σ(*F*^2^)], *wR*(*F*^2^), *S*	0.041, 0.113, 1.02
No. of reflections	4414
No. of parameters	331
H-atom treatment	H atoms treated by a mixture of independent and constrained refinement
Δρ_max_, Δρ_min_ (e Å^−3^)	0.97, −0.60

Computer programs: *PROCESS-AUTO* (Rigaku, 1998 ▸), *SIR2004* (Burla *et al.*, 2007 ▸), *SHELXL2019/2* (Sheldrick, 2015 ▸) and *ORTEPIII* (Burnett & Johnson, 1996 ▸).

## supplementary crystallographic information

### (2,7-Diethoxynaphthalene-1,8-diyl)bis[(4-bromophenyl)methanone] Crystal data

**Table:**

C_28_H_22_Br_2_O_4_	*F*(000) = 1168
*M**_r_* = 582.27	*D*_x_ = 1.598 Mg m^−^^3^
Monoclinic, *P*2_1_/*c*	Cu *K*α radiation, λ = 1.54187 Å
*a* = 7.74560 (14) Å	Cell parameters from 38566 reflections
*b* = 24.4234 (4) Å	θ = 3.5–68.3°
*c* = 13.0590 (2) Å	µ = 4.52 mm^−^^1^
β = 101.6109 (9)°	*T* = 193 K
*V* = 2419.86 (8) Å^3^	Platelet, colorless
*Z* = 4	0.60 × 0.30 × 0.10 mm

### (2,7-Diethoxynaphthalene-1,8-diyl)bis[(4-bromophenyl)methanone] Data collection

**Table:**

Rigaku R-AXIS RAPID diffractometer	4038 reflections with *I* > 2σ(*I*)
Detector resolution: 10.000 pixels mm^-1^	*R*_int_ = 0.080
ω scans	θ_max_ = 68.3°, θ_min_ = 3.6°
Absorption correction: numerical (NUMABS; Rigaku, 1999)	*h* = −9→8
*T*_min_ = 0.260, *T*_max_ = 0.636	*k* = −29→29
43148 measured reflections	*l* = −15→15
4414 independent reflections	

### (2,7-Diethoxynaphthalene-1,8-diyl)bis[(4-bromophenyl)methanone] Refinement

**Table:**

Refinement on *F*^2^	0 restraints
Least-squares matrix: full	Hydrogen site location: mixed
*R*[*F*^2^ > 2σ(*F*^2^)] = 0.041	H atoms treated by a mixture of independent and constrained refinement
*wR*(*F*^2^) = 0.113	*w* = 1/[σ^2^(*F*_o_^2^) + (0.0674*P*)^2^ + 1.6111*P*] where *P* = (*F*_o_^2^ + 2*F*_c_^2^)/3
*S* = 1.02	(Δ/σ)_max_ = 0.003
4414 reflections	Δρ_max_ = 0.97 e Å^−^^3^
331 parameters	Δρ_min_ = −0.60 e Å^−^^3^

### (2,7-Diethoxynaphthalene-1,8-diyl)bis[(4-bromophenyl)methanone] Special details

**Table:**

Geometry. All esds (except the esd in the dihedral angle between two l.s. planes) are estimated using the full covariance matrix. The cell esds are taken into account individually in the estimation of esds in distances, angles and torsion angles; correlations between esds in cell parameters are only used when they are defined by crystal symmetry. An approximate (isotropic) treatment of cell esds is used for estimating esds involving l.s. planes.

### (2,7-Diethoxynaphthalene-1,8-diyl)bis[(4-bromophenyl)methanone] Fractional atomic coordinates and isotropic or equivalent isotropic displacement parameters (Å^2^)

**Table:**

	*x*	*y*	*z*	*U*_iso_*/*U*_eq_	
Br1	0.96576 (4)	1.03404 (2)	0.36431 (3)	0.05270 (14)	
Br2	−0.39930 (5)	0.96823 (2)	−0.11254 (3)	0.05806 (14)	
O1	0.5457 (2)	0.83055 (7)	0.50431 (13)	0.0390 (4)	
O2	0.0695 (2)	0.75946 (7)	−0.00888 (13)	0.0421 (4)	
O3	0.2072 (2)	0.88406 (7)	0.30927 (14)	0.0383 (4)	
O4	0.3968 (2)	0.85776 (7)	0.11471 (13)	0.0366 (4)	
C1	0.3978 (3)	0.80814 (9)	0.33638 (17)	0.0287 (5)	
C2	0.4865 (3)	0.79004 (10)	0.43375 (18)	0.0318 (5)	
C3	0.5069 (3)	0.73420 (10)	0.45840 (19)	0.0343 (5)	
H3	0.573259	0.722794	0.524202	0.041*	
C4	0.4296 (3)	0.69638 (10)	0.38604 (19)	0.0348 (5)	
H4	0.439489	0.658560	0.403185	0.042*	
C5	0.2521 (3)	0.67269 (9)	0.2143 (2)	0.0351 (5)	
H5	0.260856	0.635155	0.233735	0.042*	
C6	0.1602 (3)	0.68654 (10)	0.1181 (2)	0.0362 (5)	
H6	0.102926	0.659242	0.071504	0.043*	
C7	0.1508 (3)	0.74268 (10)	0.08802 (18)	0.0329 (5)	
C8	0.2318 (3)	0.78325 (9)	0.15547 (18)	0.0307 (5)	
C9	0.3223 (3)	0.76908 (9)	0.25877 (17)	0.0282 (5)	
C10	0.3356 (3)	0.71244 (9)	0.28667 (19)	0.0315 (5)	
C11	0.6426 (3)	0.81596 (11)	0.60611 (18)	0.0362 (5)	
H11A	0.750029	0.795168	0.600616	0.043*	
H11B	0.569300	0.793170	0.643391	0.043*	
C12	0.6904 (5)	0.86904 (13)	0.6630 (2)	0.0522 (7)	
C13	−0.0250 (3)	0.72077 (10)	−0.08191 (18)	0.0354 (5)	
H13A	−0.112423	0.700710	−0.050432	0.043*	
H13B	0.057209	0.693956	−0.102999	0.043*	
C14	−0.1154 (4)	0.75342 (13)	−0.1744 (2)	0.0413 (6)	
C15	0.3600 (3)	0.86834 (9)	0.32537 (17)	0.0295 (5)	
C16	0.5085 (3)	0.90805 (9)	0.33684 (17)	0.0288 (5)	
C17	0.4838 (4)	0.96148 (10)	0.3671 (2)	0.0356 (6)	
H17	0.372901	0.972407	0.380858	0.043*	
C18	0.6210 (3)	0.99900 (10)	0.3774 (2)	0.0397 (6)	
H18	0.606068	1.035412	0.399668	0.048*	
C19	0.7794 (3)	0.98247 (10)	0.35463 (19)	0.0354 (5)	
C20	0.8064 (3)	0.92963 (10)	0.3238 (2)	0.0379 (5)	
H20	0.916284	0.919065	0.308167	0.046*	
C21	0.6708 (3)	0.89269 (10)	0.31622 (19)	0.0346 (5)	
H21	0.688291	0.855973	0.296535	0.042*	
C22	0.2491 (3)	0.83933 (9)	0.11044 (17)	0.0306 (5)	
C23	0.0907 (3)	0.87051 (9)	0.05858 (18)	0.0316 (5)	
C24	0.1076 (4)	0.90948 (10)	−0.0163 (2)	0.0393 (6)	
H24	0.219855	0.916307	−0.032517	0.047*	
C25	−0.0379 (4)	0.93838 (11)	−0.0673 (2)	0.0450 (6)	
H25	−0.027117	0.964235	−0.120001	0.054*	
C26	−0.1990 (4)	0.92916 (10)	−0.04062 (19)	0.0397 (6)	
C27	−0.2194 (4)	0.89222 (10)	0.0362 (2)	0.0389 (6)	
H27	−0.330361	0.887427	0.055432	0.047*	
C28	−0.0730 (3)	0.86228 (10)	0.08457 (19)	0.0359 (5)	
H28	−0.084796	0.835830	0.136056	0.043*	
H12A	0.755 (6)	0.8901 (17)	0.620 (3)	0.087 (13)*	
H12B	0.767 (5)	0.8628 (16)	0.729 (3)	0.077 (11)*	
H12C	0.582 (6)	0.8867 (16)	0.675 (3)	0.076 (12)*	
H14A	−0.198 (4)	0.7801 (14)	−0.161 (3)	0.054 (9)*	
H14B	−0.175 (5)	0.7325 (17)	−0.223 (3)	0.067 (11)*	
H14C	−0.028 (5)	0.7722 (14)	−0.202 (3)	0.060 (9)*	

### (2,7-Diethoxynaphthalene-1,8-diyl)bis[(4-bromophenyl)methanone] Atomic displacement parameters (Å^2^)

**Table:**

	*U*^11^	*U*^22^	*U*^33^	*U*^12^	*U*^13^	*U*^23^
Br1	0.0453 (2)	0.03872 (19)	0.0717 (2)	−0.01329 (11)	0.00611 (17)	0.00497 (12)
Br2	0.0582 (3)	0.0595 (2)	0.0506 (2)	0.02283 (14)	−0.00315 (16)	0.00419 (13)
O1	0.0469 (11)	0.0367 (9)	0.0299 (8)	−0.0034 (7)	−0.0007 (7)	0.0020 (7)
O2	0.0493 (11)	0.0352 (9)	0.0360 (9)	−0.0048 (8)	−0.0054 (8)	−0.0029 (7)
O3	0.0252 (9)	0.0425 (9)	0.0469 (10)	0.0039 (7)	0.0071 (7)	−0.0026 (7)
O4	0.0310 (10)	0.0418 (9)	0.0377 (9)	−0.0062 (7)	0.0085 (7)	−0.0011 (7)
C1	0.0221 (12)	0.0327 (11)	0.0327 (11)	−0.0016 (9)	0.0086 (9)	0.0015 (9)
C2	0.0254 (12)	0.0374 (12)	0.0328 (11)	−0.0029 (9)	0.0065 (9)	−0.0003 (9)
C3	0.0318 (13)	0.0382 (12)	0.0327 (12)	−0.0005 (10)	0.0056 (10)	0.0055 (9)
C4	0.0302 (13)	0.0338 (12)	0.0409 (13)	0.0009 (10)	0.0084 (10)	0.0061 (10)
C5	0.0339 (13)	0.0271 (11)	0.0453 (13)	−0.0013 (9)	0.0106 (11)	−0.0005 (9)
C6	0.0330 (14)	0.0310 (12)	0.0439 (13)	−0.0036 (10)	0.0064 (10)	−0.0055 (10)
C7	0.0281 (13)	0.0351 (12)	0.0350 (12)	−0.0013 (9)	0.0048 (10)	−0.0001 (9)
C8	0.0259 (12)	0.0313 (11)	0.0352 (12)	0.0001 (9)	0.0067 (9)	−0.0007 (9)
C9	0.0206 (12)	0.0311 (11)	0.0339 (12)	−0.0004 (8)	0.0080 (9)	−0.0001 (8)
C10	0.0256 (12)	0.0327 (12)	0.0375 (12)	0.0003 (9)	0.0091 (9)	0.0009 (9)
C11	0.0301 (13)	0.0476 (14)	0.0291 (11)	−0.0005 (10)	0.0022 (10)	0.0016 (10)
C12	0.055 (2)	0.0529 (17)	0.0416 (15)	−0.0069 (14)	−0.0068 (14)	−0.0029 (13)
C13	0.0327 (13)	0.0400 (13)	0.0338 (12)	−0.0068 (10)	0.0072 (10)	−0.0075 (10)
C14	0.0359 (15)	0.0530 (16)	0.0345 (13)	−0.0089 (13)	0.0062 (11)	−0.0027 (11)
C15	0.0262 (13)	0.0355 (12)	0.0269 (10)	0.0018 (9)	0.0059 (9)	−0.0022 (8)
C16	0.0270 (12)	0.0307 (11)	0.0277 (10)	0.0024 (9)	0.0029 (9)	0.0011 (8)
C17	0.0323 (14)	0.0342 (13)	0.0406 (14)	0.0046 (9)	0.0086 (11)	−0.0010 (9)
C18	0.0422 (15)	0.0286 (11)	0.0471 (14)	0.0029 (10)	0.0061 (11)	−0.0024 (10)
C19	0.0324 (13)	0.0320 (11)	0.0389 (13)	−0.0049 (10)	0.0001 (10)	0.0056 (10)
C20	0.0273 (13)	0.0363 (13)	0.0498 (14)	0.0010 (10)	0.0068 (10)	−0.0005 (10)
C21	0.0294 (13)	0.0304 (11)	0.0436 (13)	0.0024 (9)	0.0067 (10)	−0.0019 (9)
C22	0.0323 (13)	0.0335 (11)	0.0264 (11)	−0.0034 (9)	0.0068 (9)	−0.0030 (9)
C23	0.0331 (13)	0.0307 (11)	0.0301 (11)	−0.0013 (9)	0.0044 (9)	−0.0020 (9)
C24	0.0410 (15)	0.0389 (13)	0.0399 (13)	−0.0007 (11)	0.0124 (11)	0.0044 (10)
C25	0.0573 (18)	0.0395 (13)	0.0396 (14)	0.0059 (12)	0.0129 (12)	0.0093 (11)
C26	0.0428 (15)	0.0376 (13)	0.0355 (12)	0.0097 (11)	0.0001 (11)	−0.0015 (10)
C27	0.0330 (14)	0.0442 (13)	0.0386 (13)	0.0004 (11)	0.0051 (10)	−0.0018 (10)
C28	0.0351 (14)	0.0384 (12)	0.0337 (12)	−0.0024 (10)	0.0057 (10)	0.0042 (10)

### (2,7-Diethoxynaphthalene-1,8-diyl)bis[(4-bromophenyl)methanone] Geometric parameters (Å, º)

**Table:**

Br1—C19	1.901 (2)	C12—H12C	0.99 (4)
Br2—C26	1.901 (3)	C13—C14	1.498 (4)
O1—C2	1.367 (3)	C13—H13A	0.9900
O1—C11	1.434 (3)	C13—H13B	0.9900
O2—C7	1.358 (3)	C14—H14A	0.95 (4)
O2—C13	1.434 (3)	C14—H14B	0.87 (4)
O3—C15	1.221 (3)	C14—H14C	0.94 (4)
O4—C22	1.220 (3)	C15—C16	1.489 (3)
C1—C2	1.390 (3)	C16—C17	1.388 (3)
C1—C9	1.428 (3)	C16—C21	1.389 (3)
C1—C15	1.501 (3)	C17—C18	1.390 (4)
C2—C3	1.403 (3)	C17—H17	0.9500
C3—C4	1.370 (3)	C18—C19	1.379 (4)
C3—H3	0.9500	C18—H18	0.9500
C4—C10	1.410 (3)	C19—C20	1.381 (3)
C4—H4	0.9500	C20—C21	1.373 (3)
C5—C6	1.357 (4)	C20—H20	0.9500
C5—C10	1.417 (3)	C21—H21	0.9500
C5—H5	0.9500	C22—C23	1.486 (3)
C6—C7	1.424 (3)	C23—C24	1.389 (3)
C6—H6	0.9500	C23—C28	1.392 (4)
C7—C8	1.389 (3)	C24—C25	1.382 (4)
C8—C9	1.432 (3)	C24—H24	0.9500
C8—C22	1.507 (3)	C25—C26	1.380 (4)
C9—C10	1.429 (3)	C25—H25	0.9500
C11—C12	1.503 (4)	C26—C27	1.382 (4)
C11—H11A	0.9900	C27—C28	1.390 (4)
C11—H11B	0.9900	C27—H27	0.9500
C12—H12A	0.97 (5)	C28—H28	0.9500
C12—H12B	0.96 (4)		
			
C2—O1—C11	119.16 (19)	H13A—C13—H13B	108.7
C7—O2—C13	119.96 (19)	C13—C14—H14A	116 (2)
C2—C1—C9	119.5 (2)	C13—C14—H14B	112 (3)
C2—C1—C15	116.8 (2)	H14A—C14—H14B	105 (3)
C9—C1—C15	122.8 (2)	C13—C14—H14C	108 (2)
O1—C2—C1	115.1 (2)	H14A—C14—H14C	107 (3)
O1—C2—C3	122.8 (2)	H14B—C14—H14C	109 (3)
C1—C2—C3	122.1 (2)	O3—C15—C16	121.0 (2)
C4—C3—C2	118.9 (2)	O3—C15—C1	119.2 (2)
C4—C3—H3	120.5	C16—C15—C1	119.8 (2)
C2—C3—H3	120.5	C17—C16—C21	119.4 (2)
C3—C4—C10	121.4 (2)	C17—C16—C15	119.5 (2)
C3—C4—H4	119.3	C21—C16—C15	121.0 (2)
C10—C4—H4	119.3	C16—C17—C18	120.0 (2)
C6—C5—C10	122.1 (2)	C16—C17—H17	120.0
C6—C5—H5	119.0	C18—C17—H17	120.0
C10—C5—H5	119.0	C19—C18—C17	118.9 (2)
C5—C6—C7	118.9 (2)	C19—C18—H18	120.6
C5—C6—H6	120.5	C17—C18—H18	120.6
C7—C6—H6	120.5	C18—C19—C20	122.0 (2)
O2—C7—C8	116.2 (2)	C18—C19—Br1	119.22 (18)
O2—C7—C6	122.4 (2)	C20—C19—Br1	118.8 (2)
C8—C7—C6	121.4 (2)	C21—C20—C19	118.5 (2)
C7—C8—C9	119.9 (2)	C21—C20—H20	120.8
C7—C8—C22	117.7 (2)	C19—C20—H20	120.8
C9—C8—C22	121.5 (2)	C20—C21—C16	121.2 (2)
C1—C9—C10	117.9 (2)	C20—C21—H21	119.4
C1—C9—C8	124.1 (2)	C16—C21—H21	119.4
C10—C9—C8	118.1 (2)	O4—C22—C23	120.8 (2)
C4—C10—C5	120.3 (2)	O4—C22—C8	118.3 (2)
C4—C10—C9	120.1 (2)	C23—C22—C8	120.9 (2)
C5—C10—C9	119.6 (2)	C24—C23—C28	119.3 (2)
O1—C11—C12	106.0 (2)	C24—C23—C22	119.1 (2)
O1—C11—H11A	110.5	C28—C23—C22	121.6 (2)
C12—C11—H11A	110.5	C25—C24—C23	120.4 (2)
O1—C11—H11B	110.5	C25—C24—H24	119.8
C12—C11—H11B	110.5	C23—C24—H24	119.8
H11A—C11—H11B	108.7	C26—C25—C24	119.1 (2)
C11—C12—H12A	106 (2)	C26—C25—H25	120.5
C11—C12—H12B	111 (2)	C24—C25—H25	120.5
H12A—C12—H12B	108 (3)	C25—C26—C27	122.1 (2)
C11—C12—H12C	109 (2)	C25—C26—Br2	118.88 (19)
H12A—C12—H12C	114 (3)	C27—C26—Br2	119.0 (2)
H12B—C12—H12C	109 (3)	C26—C27—C28	118.1 (2)
O2—C13—C14	106.2 (2)	C26—C27—H27	120.9
O2—C13—H13A	110.5	C28—C27—H27	120.9
C14—C13—H13A	110.5	C27—C28—C23	120.9 (2)
O2—C13—H13B	110.5	C27—C28—H28	119.6
C14—C13—H13B	110.5	C23—C28—H28	119.6
			
C11—O1—C2—C1	−178.5 (2)	C2—C1—C15—O3	−114.3 (2)
C11—O1—C2—C3	4.0 (3)	C9—C1—C15—O3	54.9 (3)
C9—C1—C2—O1	−176.65 (19)	C2—C1—C15—C16	64.1 (3)
C15—C1—C2—O1	−7.0 (3)	C9—C1—C15—C16	−126.6 (2)
C9—C1—C2—C3	0.9 (3)	O3—C15—C16—C17	23.4 (3)
C15—C1—C2—C3	170.5 (2)	C1—C15—C16—C17	−155.0 (2)
O1—C2—C3—C4	174.1 (2)	O3—C15—C16—C21	−155.7 (2)
C1—C2—C3—C4	−3.3 (4)	C1—C15—C16—C21	25.9 (3)
C2—C3—C4—C10	2.2 (4)	C21—C16—C17—C18	−0.5 (4)
C10—C5—C6—C7	1.5 (4)	C15—C16—C17—C18	−179.5 (2)
C13—O2—C7—C8	−176.9 (2)	C16—C17—C18—C19	1.5 (4)
C13—O2—C7—C6	5.8 (4)	C17—C18—C19—C20	−1.1 (4)
C5—C6—C7—O2	176.3 (2)	C17—C18—C19—Br1	178.12 (18)
C5—C6—C7—C8	−0.8 (4)	C18—C19—C20—C21	−0.3 (4)
O2—C7—C8—C9	−179.3 (2)	Br1—C19—C20—C21	−179.53 (19)
C6—C7—C8—C9	−2.0 (4)	C19—C20—C21—C16	1.3 (4)
O2—C7—C8—C22	−10.2 (3)	C17—C16—C21—C20	−1.0 (4)
C6—C7—C8—C22	167.1 (2)	C15—C16—C21—C20	178.1 (2)
C2—C1—C9—C10	2.5 (3)	C7—C8—C22—O4	−118.5 (2)
C15—C1—C9—C10	−166.5 (2)	C9—C8—C22—O4	50.5 (3)
C2—C1—C9—C8	−178.6 (2)	C7—C8—C22—C23	59.3 (3)
C15—C1—C9—C8	12.5 (3)	C9—C8—C22—C23	−131.8 (2)
C7—C8—C9—C1	−175.0 (2)	O4—C22—C23—C24	23.3 (3)
C22—C8—C9—C1	16.3 (3)	C8—C22—C23—C24	−154.3 (2)
C7—C8—C9—C10	4.0 (3)	O4—C22—C23—C28	−155.5 (2)
C22—C8—C9—C10	−164.7 (2)	C8—C22—C23—C28	26.8 (3)
C3—C4—C10—C5	−178.0 (2)	C28—C23—C24—C25	−2.4 (4)
C3—C4—C10—C9	1.2 (4)	C22—C23—C24—C25	178.8 (2)
C6—C5—C10—C4	179.8 (2)	C23—C24—C25—C26	1.9 (4)
C6—C5—C10—C9	0.5 (4)	C24—C25—C26—C27	0.5 (4)
C1—C9—C10—C4	−3.5 (3)	C24—C25—C26—Br2	−179.1 (2)
C8—C9—C10—C4	177.4 (2)	C25—C26—C27—C28	−2.5 (4)
C1—C9—C10—C5	175.8 (2)	Br2—C26—C27—C28	177.15 (18)
C8—C9—C10—C5	−3.3 (3)	C26—C27—C28—C23	2.0 (4)
C2—O1—C11—C12	179.0 (2)	C24—C23—C28—C27	0.4 (4)
C7—O2—C13—C14	173.3 (2)	C22—C23—C28—C27	179.2 (2)

### (2,7-Diethoxynaphthalene-1,8-diyl)bis[(4-bromophenyl)methanone] Hydrogen-bond geometry (Å, º)

*Cg*2, *Cg*3 and *Cg*5 are the centroids of the C5–C10, C16–C21 and C1–C10 rings, respectively.

**Table:**

*D*—H···*A*	*D*—H	H···*A*	*D*···*A*	*D*—H···*A*
C20—H20···O3^i^	0.95	2.41	3.339 (3)	167
C27—H27···O4^ii^	0.95	2.50	3.439 (3)	170
C11—H11*B*···*Cg*2^iii^	0.99	2.75	3.632 (3)	148
C11—H13*B*···*Cg*5^iii^	0.99	2.89	3.838 (3)	162
C14—H14*C*···*Cg*2^iv^	0.98	2.78 (4)	3.631 (3)	151 (3)
C14—H14*C*···*Cg*5^iv^	0.98	2.85 (4)	3.632 (3)	141 (3)
C12—H12*A*···Br1^v^	0.97 (4)	2.82 (5)	3.634 (4)	141 (3)
C26—Br2···*Cg*3^vi^	1.90 (1)	3.88 (1)	5.626 (3)	152 (1)

Symmetry codes: (i) *x*+1, *y*, *z*; (ii) *x*−1, *y*, *z*; (iii) *x*, −*y*+3/2, *z*+1/2; (iv) *x*, −*y*+3/2, *z*−1/2; (v) −*x*+2, −*y*+2, −*z*+1; (vi) −*x*, −*y*+2, −*z*.
