# Supplementary material for: Crystal structure and Hirshfeld surface analysis of (2,7-di­eth­oxy­naphthalene-1,8-di­yl)bis­[(4-bromophen­yl)methanone]
Source: Acta Crystallogr E Crystallogr Commun. 2024 Nov 22;80(Pt 12):1313–7. doi: 10.1107/S205698902401123X (PMC11789185; doi:10.1107/S205698902401123X)
Supplement: Supplementary file 3 [file e-80-01313-sup3.pdf]

Supporting information for

Crystal structure and Hirshfeld surface analysis of (2,7-diethoxynaphthalene-1,8-diyl)bis((4-bromophenyl)methanone)

Kun Li, Jiali Yao, Hiroaki Iitsuka, Noriyuki Yonezawa, and Akiko Okamoto*
Department of Organic and Polymer Materials Chemistry
Tokyo University of Agriculture and Technology
2-24-16 Nakamachi, Koganei, Tokyo, Japan

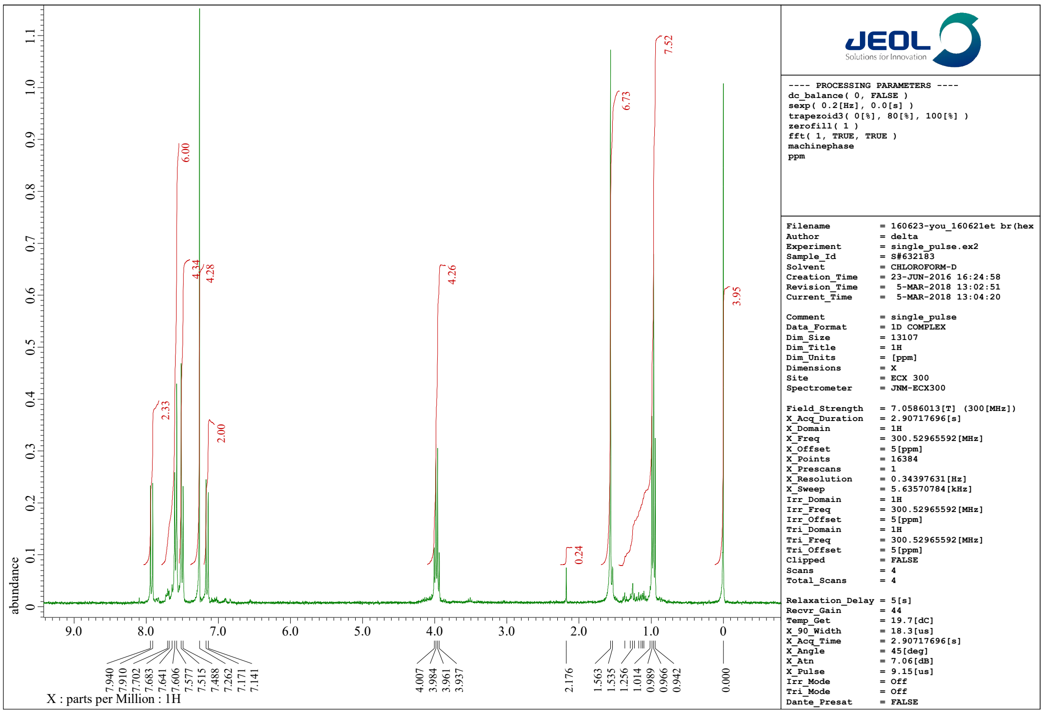

You created this PDF from an application that is not licensed to print to novaPDF printer (http://www.novapdf.com)

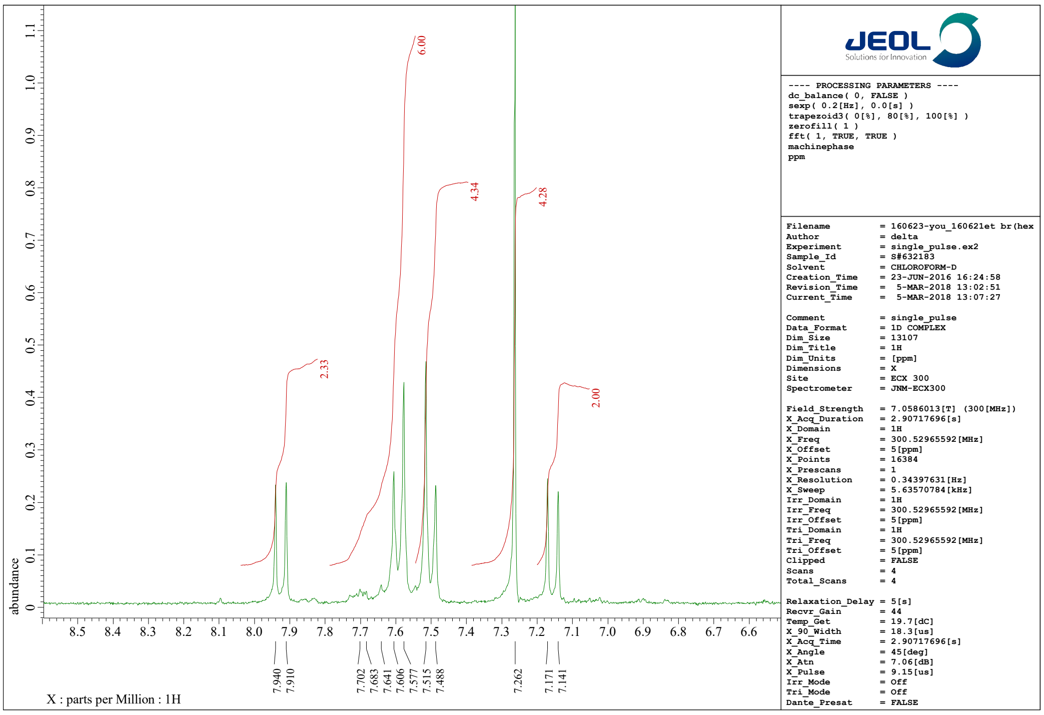

You created this PDF from an application that is not licensed to print to novaPDF printer (http://www.novapdf.com)

Figure S-1. ^{1}H NMR spectra of title compound.

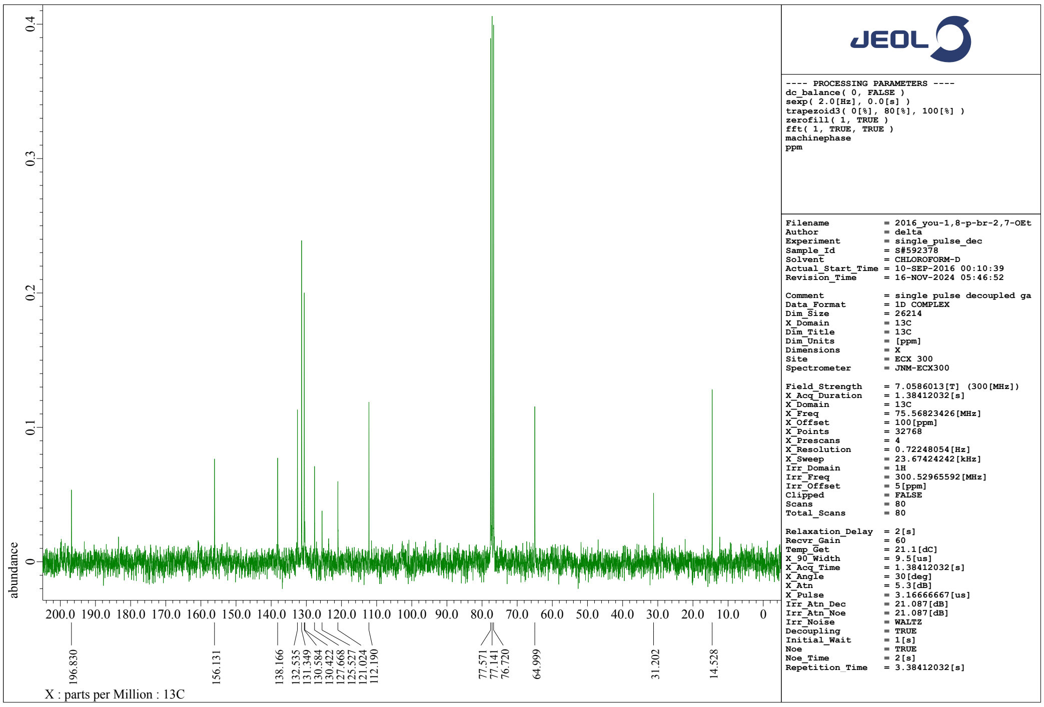

Figure S-2. ^{13}C NMR spectrum of title compound.

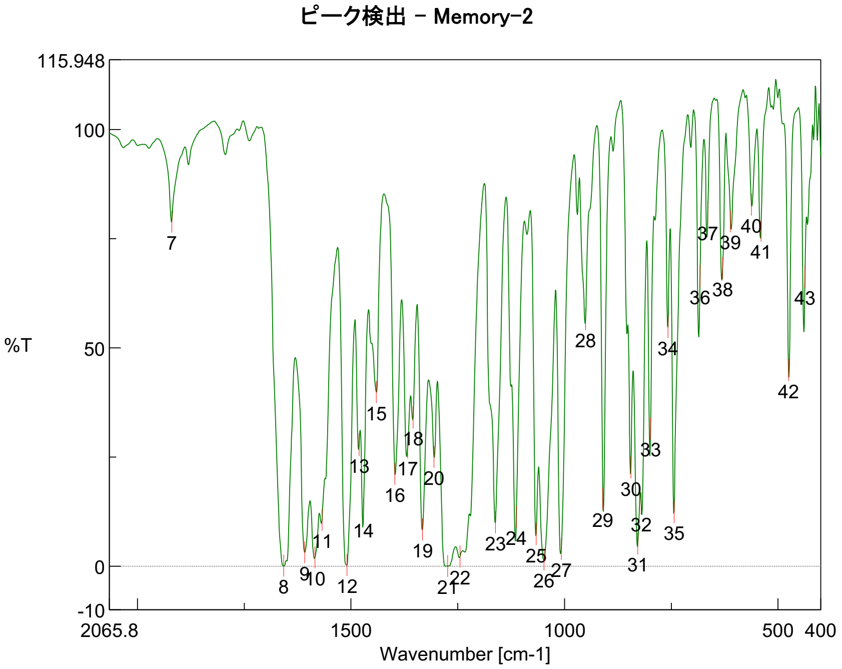

[コメント情報]

試料名	1,8-Br-2,7-oEt
コメント	
測定者	
所属	
会社	東京農工大学

[データ情報]

作成日時	2016/09/29 10:51
データタイプ	等間隔データ
横軸	Wavenumber [cm-1]
縦軸	%T
スタート	399.193 cm-1
エンド	4000.6 cm-1
データ間隔	0.964233 cm-1
データ数	3736

[測定情報]

機種名	FT/IR-4100typeA
シリアル番号	B041461016
測定日時	2016/07/29 16:53
光源	標準光源
検出器	TGS
積算回数	16
分解	4 cm-1
ゼロフィリング	On
アポダイゼーション	Cosine
ゲイン	Auto (32)
アパーチャー	Auto (7.1 mm)
スキャンスピード	Auto (2 mm/sec)
フィルタ	Auto (30000 Hz)

[ピーク検出結果]

No.	位置	強度	No.	位置	強度
1	3088.44	77.9363	2	3057.58	81.7665
3	3031.55	90.2977	4	2979.48	40.6495
5	2932.23	53.8929	6	2885.95	55.8639
7	1919.79	78.9168	8	1657.52	0.090631
9	1608.34	3.16759	10	1584.24	2.02001
11	1567.84	10.5936	12	1509.03	0.265075
13	1481.06	27.7514	14	1470.46	12.9341

Figure S-3. IR spectrum of title compound.
